# The mutational landscape of melanoma brain metastases presenting as the first visceral site of recurrence

**DOI:** 10.1038/s41416-020-01090-2

**Published:** 2020-10-07

**Authors:** Roy Rabbie, Peter Ferguson, Kim Wong, Dominique-Laurent Couturier, Una Moran, Clinton Turner, Patrick Emanuel, Kerstin Haas, Jodi M. Saunus, Morgan R. Davidson, Sunil R. Lakhani, Brindha Shivalingam, Georgina V. Long, Christine Parkinson, Iman Osman, Richard A. Scolyer, Pippa Corrie, David J. Adams

**Affiliations:** 1 Experimental Cancer Genetics, The Wellcome Sanger Institute, Hinxton, Cambridgeshire UK; 2 Cambridge Cancer Centre, Cambridge University Hospitals NHS Foundation Trust, Cambridge, UK; 3 Melanoma Institute Australia, The University of Sydney, Sydney, NSW 2065 Australia; 4 Sydney Medical School, The University of Sydney, Sydney, NSW 2006 Australia; 5 Royal Prince Alfred Hospital, Camperdown, NSW 2050 Australia; 6 Cancer Research UK Cambridge Institute, University of Cambridge, Li Ka Shing Centre, Robinson Way, Cambridge, UK; 7 Interdisciplinary Melanoma Program, New York University School of Medicine, New York, NY USA; 8 Anatomical Pathology, LabPLUS Auckland City Hospital, Auckland, New Zealand; 9 Anatomic Pathology, The University of Auckland, Auckland, New Zealand; 10 Cancer Genomics Laboratory, Francis Crick Institute, London, UK; 11 UQ Centre for Clinical Research, The University of Queensland Faculty of Medicine, Herston, QLD Australia; 12 Pathology Queensland, Royal Brisbane Women’s Hospital, Herston, QLD Australia; 13 Royal North Shore and Mater Hospitals, Sydney, NSW 2065 Australia

**Keywords:** CNS cancer, Metastasis, Melanoma, Tumour biomarkers, Cancer

## Abstract

Brain metastases are a major cause of melanoma-related mortality and morbidity. We undertook whole-exome sequencing of 50 tumours from patients undergoing surgical resection of brain metastases presenting as the first site of visceral disease spread and validated our findings in an independent dataset of 18 patients. Brain metastases had a similar driver mutational landscape to cutaneous melanomas in TCGA. However, *KRAS* was the most significantly enriched driver gene, with 4/50 (8%) of brain metastases harbouring non-synonymous mutations. Hotspot *KRAS* mutations were mutually exclusive from *BRAF*^*V600*^, *NRAS* and *HRAS* mutations and were associated with a reduced overall survival from the resection of brain metastases (HR 10.01, *p* = 0.001). Mutations in *KRAS* were clonal and concordant with extracranial disease, suggesting that these mutations are likely present within the primary. Our analyses suggest that *KRAS* mutations could help identify patients with primary melanoma at higher risk of brain metastases who may benefit from more intensive, protracted surveillance.

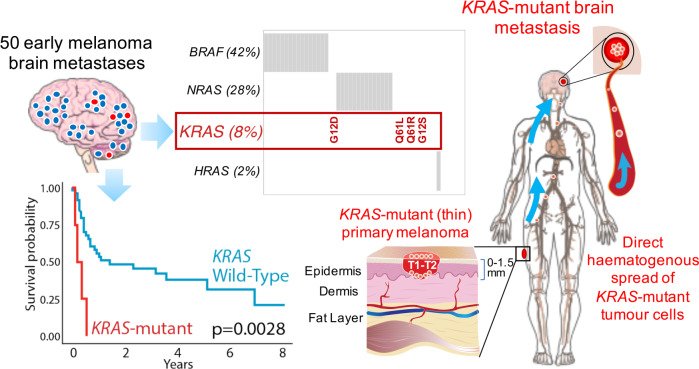

## Background

Metastases to the central nervous system (CNS) are observed in ~60% of cutaneous melanoma patients developing disseminated disease and up to 90% at autopsy.^1^ Early detection of intracerebral recurrence remains critical, as isolated or oligometastatic brain metastases may be more amenable to potentially curative locoregional therapies and immunotherapies have demonstrated greatest efficacy in patients with small, asymptomatic metastases.^1,2^ Early predictors of brain metastases could therefore help identify those patients most likely to benefit from closer surveillance of the brain as well as inform early use of adjuvant therapies. Importantly, epidemiological data suggest that patterns of metastatic dissemination may be partially determined by the clinical characteristics of the primary tumour.^3^

Interestingly, 15–20% of brain metastases present as the isolated first visceral site of disease spread.^4^ Primary tumours in these ‘early brain metastasis’ cases were reported as thinner and of lower American Joint Committee of Cancer Stage when compared to other visceral metastases, challenging the current understanding of brain metastases as the final stage of tumour progression, and suggesting that these tumours could harbour distinct biological properties favouring early haematogenous dissemination to the brain.^4^ Our analyses of the mutational landscape of early brain metastasis highlights key molecular features that could inform future prognostic, surveillance and intervention strategies.

## Methods

### Study population

Patients with available archival paraffin-embedded melanoma brain metastases (in the absence of other sites of visceral disease, confirmed by computed tomography or magnetic resonance imaging prior to neurosurgery) were selected from prospectively maintained databases at The Melanoma Institute of Australia (*n* = 34), The Wellington School of Medicine (*n* = 8), New York University School of Medicine (*n* = 4) and Cambridge University Hospitals (*n* = 4) (discovery cohort). Samples from patients selected from The University of Queensland Australia and the Auckland region New Zealand (*n* = 18 total) made up the external validation cohort. All neuro-resections were undertaken between 2008 and 2018 at the respective academic neurosurgical centres as part of routine clinical care. All cases were ethically approved by the local Institutional Review Boards, as well as by the Sanger Institute’s human materials and data management committee. All samples and clinical details are listed in Supplementary Table 1.

The clinical and mutation data from The Cancer Genome Atlas (SKCM-TCGA)^5^ was downloaded from the cBioPortal. The melanoma cases from the Memorial Sloan Kettering MSK-IMPACT data set (SKCM-MSK-IMPACT) were extracted from the publication by Zehir et al.^6^ (Supplementary Methods and Supplementary Table 2).

### DNA sequencing

Exome capture of the discovery cohort was performed using Agilent SureSelect All Exon V5 baits. Paired-end sequencing was performed using the Illumina HiSeq (Illumina, San Diego, CA, USA) platform at the Wellcome Sanger Institute. MuTect (v1.1.7) and Sequenza (v2.1.2) were used to call somatic single nucleotide variants (SNVs) and copy number aberrations, respectively. Melanoma-driver SNVs called in the whole-exome-sequenced discovery cohort were orthogonally validated (with an aliquot of the same DNA) using a custom gene panel designed to capture (*n* = 287) cancer-driver genes identified from analysis of the TCGA and ICGC cohorts (Supplementary Methods and Supplementary Table 3, ELID ID: 0822402). Panel sequencing of the 18 samples in the external validation cohort was performed using custom pull-down and sequencing of 549 key melanoma and related cancer-driver genes (Supplementary Methods and Supplementary Table 4, ELID ID: 3065404).

#### Tests of equality of proportions

Wald *t* tests for logistic regression parameters were used to test the equality of mutational frequencies in the discovery cohort and the reference data sets. Similar conclusions were obtained by means of Chi-square and Fisher’s exact tests, see Supplementary Methods.

#### Survival analyses

Kaplan–Meier plots were used to compare survival of *KRAS* mutations within the discovery cohort and The Cancer Genome Atlas (SKCM-TCGA). Between-group differences in instantaneous risk were assessed by fitting univariate and multivariate Cox proportional hazards regression models and defining 95% confidence intervals (CIs) for relevant hazard ratios (HRs). Multivariate models were controlled for further predictors including sex, age, centre, *BRAF* and *NRAS* mutation status (as well as primary tumour characteristics where relevant), see Supplementary Methods for details.

## Results

Fifty patients who developed brain metastases as their first site of visceral disease spread were enrolled as part of the discovery cohort and were represented by a relatively high proportion of thin (T1–T2) (*n* = 25, 50%) and non-ulcerated (*n* = 26, 52%) primary melanomas (Supplementary Table 1).

**Fig. 1:**
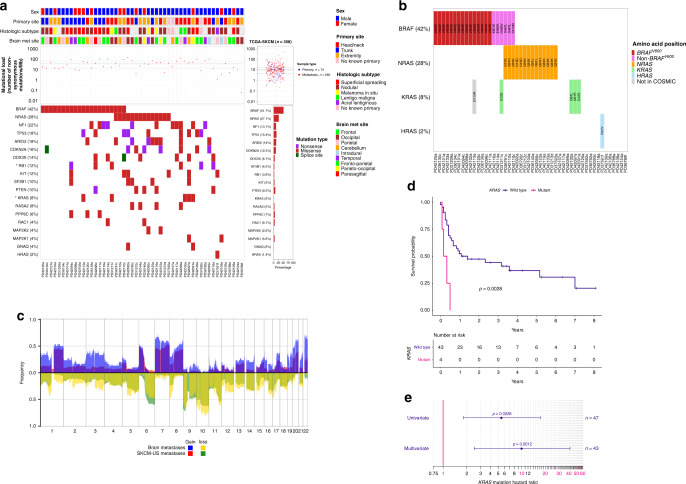
Tile plot of melanoma-driver mutations in the early brain metastasis discovery cohort (*n* = 50). **a** The mutational profiles of early brain metastases are indicated. Mutational load was calculated as the number of non-synonymous mutations per Mb, median is indicated by the solid horizontal grey line and the 95% confidence interval by the dashed lines (median 38 mutations/Mb, 95% CI 14.2–67.1). The genes shown carry non-synonymous mutations within the selected melanoma drivers outlined in Hayward et al. (*n* = 19) and are ordered according to their mutation frequency within this cohort. The corresponding mutational load and gene mutational frequencies in the SKCM-TCGA dataset (*n* = 358) are indicated. *KRAS*; 8% (4/50) in our dataset vs 2% (7/358) in the SCKM-TCGA collection, *p* = 0.0227, logistic regression Wald *t* test, *RB1*; 6/50 12% in our dataset vs 3.9% 14/358 in SKCM-TCGA, *p* = 0.019, logistic regression Wald *t* test. **b** Focussed tile plot from **a**, highlighting the mutated amino acid positions within the *RAS* signalling genes. As expected, mutations in *NRAS* were mutually exclusive to *BRAF*^*V*600^ hotspots. The four hotspot *KRAS* mutations were also mutually exclusive to *BRAF*^V600^ and to mutations in *NRAS* and *HRAS*. Note that, although we identified five KRAS mutations within the early brain metastasis discovery cohort, the *KRAS*^*G115R*^ mutation shaded in grey (occurring in association with a *BRAF*^*V600E*^-driver mutation in sample PD42113a) is exceptionally rare^7^ and was not considered pathogenic. **c** Copy number profile of the early melanoma metastasis discovery cohort (*n* = 30) overlaid onto the copy number profile of SKCM-TCGA (*n* = 337). The non-overlaid plots are shown in Supplementary Fig. 3. **d** Kaplan–Meier survival curves showing overall survival probabilities from resection of brain metastasis (defined as the time from the resection of the brain metastasis to last follow-up (right-censored) or death from any cause) as a function of time for the hotspot *KRAS*-mutant (*n* = 4) vs *KRAS* wild-type (*n* = 43) patients (3 *KRAS* wild-type patients did not have survival data available). Patients with *KRAS*-mutant tumours had significantly worse overall survival from resection of brain metastasis than *KRAS* wild-type patients, median 3 vs 12 months (*p* = 0.003, univariate Cox regression). **e** Forest plot comparing *KRAS*-mutant vs wild-type hazard ratio for survival from resection of brain metastasis in univariate (HR 5.58, 95% CI 1.80–17.24, *p* = 0.003, *n* = 47) and multivariate (HR 10.01, 95% CI 2.49–40.98, *p* = 0.0012, *n* = 43) Cox proportional hazards regression models. Multivariate correction was undertaken for gender, centre and age at resection of brain metastasis as well as *BRAF* and *NRAS* mutation status.

Mutations in *BRAF* were detected in 21 (42%) tumours, of which 15/21 (71%) were in the V600 hotspot (Fig. 1a, b). *NRAS* mutations were identified in 14 (28%) tumours and were all in hotspot positions on exons 2 (codons 12 and 13) and 3 (codon 61) and mutually exclusive from *BRAF*^*V600*^ mutations. Comparing the mutational landscape of brain metastases to that of cutaneous melanomas in the SKCM-TCGA dataset (see Supplementary Methods), *KRAS* was the most significantly enriched driver gene in our dataset, mutated in 8% (4/50) vs 2% (7/358) in the entire SCKM-TCGA collection (*p* = 0.0227, logistic regression Wald *t* test). Note that, although we identified 5 *KRAS* mutations within the discovery cohort, the *KRAS*^*G115R*^ mutation (occurring in association with a *BRAF*^*V600E*^-driver mutation in sample PD42113a) is exceptionally rare^7^ and was not considered pathogenic (Fig. 1b). The mutation frequency of *KRAS* was also significantly enriched relative to the frequency of extracranial melanoma metastases; 8% (4/50) in our dataset vs 2.1% (6/274) in extracranial melanoma metastases in SKCM-TCGA (*p* = 0.0413, logistic regression Wald *t* test, see Supplementary Methods). Further, only 1.6% (3/186) of melanoma cases in the Memorial Sloan Kettering MSK-IMPACT dataset were *KRAS* mutant, significantly lower than in our early brain metastasis discovery cohort (*p* = 0.0327, logistic regression Wald *t* test). The odds of observing a *KRAS* mutation in a given sample within the early brain metastases discovery cohort was approximately fourfold higher than in these three reference datasets (Supplementary Fig. 1). Mutations in *KRAS* had a high variant allele frequency (median 0.77 (0.50–0.86), indicating that they likely represent clonal (early) driver mutations (Supplementary Tables 5 and 6). Of note, three extracranial metastases available for sequencing from two patients with *KRAS*-mutant brain metastases also harboured the same brain-metastatic *KRAS* mutations, suggesting that *KRAS* mutations were concordant with extracranial metastases (see Supplementary Methods). Notably, mutations in *KRAS* were in hotspot codons 12 and 61 and mutually exclusive from other mutations in the mitogen-activated protein kinase (MAPK) signalling genes including *BRAF*^*V600*^, *NRAS* and *HRAS*, and this pattern of mutually exclusivity was also observed in *KRAS*-mutant melanomas within the SKCM-TCGA and SKCM-MSK datasets (Fig. 1b and Supplementary Table 6).

We conducted a further custom pull-down validation experiment on selected melanoma-driver mutations within the discovery cohort and confirmed 56/60 (93%) to be somatic mutations (see Supplementary Methods). We also conducted another external validation experiment, analysing a further 18 early metastases independently acquired from two different neurosurgical centres (see Supplementary Methods). This revealed that 1 brain metastasis (5.6%) harboured a *KRAS*^*G13C*^ mutation, which was also mutually exclusive from mutations in the RAS signalling genes (*BRAF*/*NRAS*/*HRAS*) (Supplementary Fig. 2 and Supplementary Table 5). The copy number landscape of the early brain metastases discovery cohort proved remarkably similar to that of SKCM-TCGA cohort (Fig. 1c and Supplementary Fig. 3).

All patients with *KRAS*-mutant brain metastases succumbed to disease, with a median overall survival from resection of brain metastasis of only 3 months, compared to 12 months in patients with resected *KRAS* wild-type brain metastases (HR 10.01, 95% CI 2.49–40.98, *p* = 0.0012, *n* = 43, covariate corrected Cox proportional hazards model, Fig. 1d, e and Supplementary Fig. 4). Melanoma patients with tumours harbouring *KRAS* mutations or amplifications represented in the SCKM-TCGA dataset were also associated with worse overall survival compared to *KRAS* wild-type melanomas (HR 2.59, 95% CI 1.21–5.55, *p* = 0.015, *n* = 352, univariate Cox regression), although this did not meet the threshold for statistical significance after correction of clinical covariates likely due to the limited sample size (HR 2.04, 95% CI 0.88–4.75, *p* = 0.098, *n* = 322, multivariate corrected Cox proportional hazards regression, Supplementary Fig. 5 and Supplementary Table 2).

## Discussion

This analysis represents the largest survey of mutation profiles of melanoma brain metastases. Consistent with the landmark melanoma sequencing studies (primarily based on extracranial metastases),^5,6^ early melanoma brain metastases were dominated by a high mutational burden (with a predominance of C > T nucleotide transitions at dipyrimidines) and a similar frequency of the key driver mutations, including *BRAF* (42%), *NRAS* (28%), *NF1* (22%) and *TP53* (18%). This is the first study to show significant enrichment of *KRAS* mutations in melanoma brain metastases as well as an association of *KRAS* mutations with adverse outcomes. The predominance of *KRAS* mutations in codons 12, 13 and 61 as well as the mutual exclusivity with other key drivers of MAPK signalling suggests that these likely represent important drivers in this context.

The RAS family of GTPases consists of genes including *NRAS*, *KRAS* and *HRAS* mutated in 25, 2 and 1% of melanomas, respectively.^5^ *NRAS*-mutant melanomas are recognised to be more aggressive and associated with poorer outcomes; however, very little is known about *KRAS-*mutant melanoma.^8^ *KRAS*-mutant early brain metastases in our study generally emanated from thin and non-ulcerated primary melanomas (Supplementary Table 7). Hence, *KRAS* detection might in future be used to ‘upstage’ a subgroup of lower-risk patients not currently offered routine surveillance and/or adjuvant therapy potentially avoiding the devastating impact of brain metastases. Mutations in *KRAS* were clonal and concordant with extracranial disease, which suggests that these mutations are present within the primary tumour; however, further studies will be required to confirm this.

The MAPK and phosphoinositide-3 kinase (PI3K) pathways are the two key downstream signalling pathways through which constitutively activated RAS exerts its pro-tumorigenic effects. MAPK pathway activation and brain metastases are inextricably connected and *BRAF* and *NRAS* mutations are associated with an increased risk of brain metastasis.^9^ In the same way, the PI3K/AKT pathway has been mechanistically linked with the development of brain metastases and analyses of patient-matched pairs of brain and extracranial metastases have revealed that brain metastases have higher levels of activated AKT and lower expression of *PTEN*, a finding also observed using immunohistochemistry.^10^ Hotpot *KRAS* mutations are known to activate *EGFR* signalling pathways, which in-turn is associated with an increased risk of brain metastases in non-small cell lung cancer.^11^

Transgenic mouse models have established that oncogenic *Kras* can induce naevi and be a founder event in melanomagenesis.^12^ In one study, a *Kras*^*G12D*^ allele was combined with alleles of *p53* or *Lkb1* and with a melanocyte-specific Cre driver to generate a model that developed melanoma with a penetrance of 100%.^13^ In this study, metastases were identified in lymph node, lung, liver and spleen but not in kidney or brain. It is therefore important to consider that, while we observe an increased frequency of *KRAS* mutations associated with early brain metastases, it is also plausible that *KRAS* mutations may play a more general role in metastases. Well-conducted in vivo studies will be needed to further uncover the potential for site-specific metastatic tropism of specific *KRAS* variants.

The retrospective nature of this analysis could feasibly introduce a degree of selection bias, in particular by only identifying those patients with operable early brain metastasis we might have excluded a larger patient demographic with more widespread disease. Emerging evidence indicates that metastatic outgrowth may also depend on the interplay between cancer cells and the host stroma; however, such tumour-cell extrinsic factors would not be fully captured by this analysis. The identification of *KRAS* mutations as a predictive biomarker for the development of early brain metastases will ultimately require prospective validation in larger cohorts employing multivariate models, particularly assessing the predictive value of these mutations in relation to other clinical covariates.^3^

In summary, our analyses indicate that the patterns of melanoma recurrence may be at least partially determined by the tumour mutational profile and that up to 8% of patients developing early brain metastases may have tumours driven by oncogenic *KRAS* mutations. This observation has implications for deciphering the biology of site-specific metastatic pathogenesis and, if validated in larger prospectively curated cohorts, might influence prognosis, surveillance and interventions in patients carrying these somatic alterations.

## Supplementary information

Supplemental Methods and Figures

Supplementary Tables 1–7

## Data Availability

All the whole-exome and targeted sequencing data (including raw sequencing files, variant calls and copy number calls) have been deposited at the European Genome-Phenome Archive (https://www.ebi.ac.uk/ega/ at the EBI) under study accession ID EGAS00001002107.
