# Supplementary material for: The mutational landscape of melanoma brain metastases presenting as the first visceral site of recurrence
Source: Br J Cancer. 2020 Oct 7;124(1):156–60. doi: 10.1038/s41416-020-01090-2 (PMC7782512; doi:10.1038/s41416-020-01090-2)
Supplement: Supplementary file 1 — Supplemental Methods and Figures [file 41416_2020_1090_MOESM1_ESM.pdf]

The mutational landscape of melanoma brain metastases presenting as the first visceral site of recurrence

Rabbie *et al.* 2020

Supplemental Methods and Figures

Supplemental Methods

Patient enrolment

Patients with surgically resected brain metastases and no other visceral sites of metastatic disease (assessed prior to neurosurgery by CT or MRI imaging), were eligible for inclusion. Patients with available archival paraffin embedded melanoma brain metastases suitable for DNA extraction were selected from prospectively maintained databases from; The Melanoma Institute of Australia (n=34), The Wellington School of Medicine (n=8), New York University School of Medicine (n=4) and Cambridge University Hospitals (n=4) (total 50, discovery cohort). Samples from patients selected from the Auckland region and The University of Queensland Australia (9 and 9 cases respectively) made up the external validation cohort. Two additional patients (comprising 3 additional samples) from the discovery cohort with hotspot *KRAS* mutations who had available extracranial tumour tissue for molecular analysis were also sequenced for the presence of concordant extracranial *KRAS*-mutations (patient MBM_Disc_23 sample PD42097c regional lymph node, and patient MBM_Dis_40 samples PD31211d/PD31211e both extracranial skin metastases, clinical details in **Supplementary Table 1** and sequencing data in **Supplementary Data**). All neuro-resections were undertaken between 2008 and 2018 at the respective academic neurosurgical centres as part of routine clinical care. All cases were ethically approved by the local Institutional Review Boards (REC approval reference numbers; HREC/RPAH/444, 16/CEN/149, 10362, 11/NE/0312, HREC 2005/022 and 16/CEN/149 for the six centres outlined above respectively), as well as by the Sanger Institute's human materials and data management committee. All samples and clinical details are listed in **Supplementary Table 1**.

Extraction of clinical details

Patient demographics, primary tumour characteristics (date of primary diagnosis, Breslow thickness, ulceration, mitotic rate, N stage), date of diagnosis of brain metastasis, date of neurosurgical resection, sites of surgically resected brain metastases as well as date of last follow-up or death, were extracted primarily from prospectively maintained clinical research databases and, if needed, through further review of the clinical record. None of the patients received

systemic therapies (included targeted and immunotherapies) prior to the resection of brain metastases.

Extraction and quality assessment of DNA and RNA

For each tumour, a hematoxylin and eosin-stained (H&E) section was reviewed by a consultant histopathologist (PF, RAS, CT and PE) for confirmation of the histopathological diagnosis and to identify appropriate regions for DNA extraction. All samples were obtained as either 1.0 mm diameter cores or 4um tissue sections micro-dissected from the original FFPE block. Germline DNA was extracted from micro-dissected adjacent normal tissue where available (n=36). Genomic DNA was extracted using the QIAamp FFPE Tissue kit from Qiagen according to manufacturer's instructions.

Whole exome-sequencing of the discovery cohort

Exome capture was performed using the Agilent SureSelect Human All Exon V5 platform. Paired-end sequencing was performed using the Illumina HiSeq (Illumina, San Diego, CA, USA) platform at the Wellcome Sanger Institute to generate 75 bp paired-end reads. Sequencing reads were aligned using BWA-MEM (v0.7.17-r1188)[1] to the human reference genome hs37d5. PCR duplicates, secondary read alignments, and reads that failed Illumina chastity (purity) filtering were flagged and removed prior to running variant and copy number calling. For tumour samples (n=16) where no matching germline DNA was available, a panel of 39 FFPE-extracted normals was used to filter germline variants as well as artefacts. To ensure that tumour and matched normal samples had the best reciprocal genotype match, SAMtools mpileup, followed by BCFtools gtcheck were run to detect sample concordance, potential sample swaps and contamination. The resulting median sequencing coverage in the brain metastatic samples from the discovery cohort (excluding PCR duplicates) was 48x (range 11-134x) in the tumour samples and 47x (range 6-149x) in the germline samples.

MuTect (v1.1.7)[2] and Strelka (v2.9.2)[3] were used to call somatic SNVs and indels, respectively. Prior to running Strelka, Manta (1.5.0)[4] was run and candidate large indels were used as input to Strelka. The minimum base quality score for somatic and germline variant calling was set to Phred 30. The Ensembl Variant Effect Predictor[5] was used to predict the effect of variants on genes and proteins, relative to the gene build in Ensembl version 97. To remove artefacts, MuTect variant

calls were filtered using a tiered approach, such that only the SNVs that met the following criteria were reported (1) Lower coverage samples (<40x); Variant Allele Frequency (VAF) >0.1. (2) All other depth samples; requirement of at least 2 alternative bases on each strand, total depth >=5, and VAF>=0.1 Variant calls found in the gnomAD database[6] were removed if the global population variant allele frequency was greater than or equal to 0.01. Mutational load was calculated as the number of non-synonymous mutations per Mb. The alignments for all reported variants in melanoma driver genes (**Fig. 1a**) were visually inspected using JBrowse and IGV.

Of note extracranial tumour tissue was available and whole-exome sequenced for two hotpot *KRAS* mutant patients. Patient MBM_Disc_23 sample PD42097c - regional lymph node also carried a $KRAS^{Q61R}$ mutation, and patient MBM_Dis_40 samples PD31211d/PD31211e - extracranial skin metastases also carried $KRAS^{G12D}$ mutations, both concordant with the *KRAS* mutation in the matched brain metastases (raw data deposited in **Supplementary Data**).

Copy number profiling of the discovery cohort

Sequenza (v2.1.2)[7] was used to estimate tumour cellularity and ploidy from 30 tumour-normal pairs in the discovery WES cohort (36/50 discovery cohort samples were paired with germline; minus 5 samples with coverage < 20x and one sample with a noisy CNV profile PD36788e), as well as to calculate allele-specific copy number profiles. For each sample, the default best-fit solution was used for the cellularity and ploidy estimates. To call copy number gain or loss in each sample, the neutral copy number was set as the weighted mean copy number of the segments, rounded to the nearest whole number.

Targeted panel sequencing of the external validation cohort

Panel sequencing of the 18 samples in the external validation was performed using custom pull-down and sequencing of 549 key melanoma and related cancer driver genes (**Supplementary Table 3**). A custom capture probe was designed using Agilent Technologies' online software 'Sure Select Design Wizard' (ELID Design ID: 3065404). DNA capture libraries were created using native DNA (paired-end, average insert size 150bp). Libraries were multiplex sequenced using the Illumina HiSeq platform, excluding reads from PCR duplicates. Only samples with an average depth of >10x (excluding PCR duplicates) across the entire bait were included. SAMtools mpileup was used to identify non-reference bases in: *BRAF* (10 loci covering hotspot SNVs in codons: 469,

586, 594, 599, 600, 601), *NRAS* (12 loci covering hotspot SNVs in codons: 12, 13, 59, 60, 61, 146), *HRAS* (7 loci covering hotspot SNVs in codons: 12, 13, 61) and *KRAS* (15 loci covering hotspot SNVs in codons: 11, 12, 13, 61, 117, 146). Thus, providing a total of 44 loci for interrogation in each of the 18 (tumour-only) samples. Only non-reference variants (with minimum based quality 30 and mapping quality 10) supported by at least 2 alternate bases (from reads not marked as PCR duplicates) are reported. The median sequencing coverage (excluding PCR duplicate reads) across all these hotspot loci in the 18 external validation samples was 44x, with 700/792 (88%) of loci covered with ≥ 10 bases.

Orthogonal validation of SNVs in the discovery cohort

Melanoma driver single nucleotide variants (SNVs) called in the whole-exome sequenced discovery cohort were orthogonally validated (with an aliquot of the same DNA) using a custom gene panel designed to capture (n=287) cancer driver genes in solid tumours within TCGA and ICGC (**Supplementary Table 4**, ELID ID: 0822402). A variant called in the discovery cohort (through whole-exome sequencing) that was also present in the orthogonal validation custom panel (with minimum base quality 30 and mapping quality 10) and supported by at least 1 alternate bases in the validation, is reported as validated somatic. With these criteria, 56/60 (93%) of the substitutions were validated somatic (true positives) (**Supplementary Data**). The (n=4) SNVs called in the discovery cohort that were not validated in the orthogonal validation may represent subclonal mutations that require greater depth rather than false positive calls.

Comparison of the mutational profiles to cutaneous melanomas represented in TCGA and the MSK-IMPACT datasets

In order to compare the mutational rates and frequencies to our dataset (represented by one sample per patient), the clinical and mutation data from The Cancer Genome Atlas (SKCM-TCGA)[8], were downloaded from the cBioPortal[9,10]. Samples were filtered to a single sample per patient giving a total of 358 samples from 358 patients, of which all samples had appropriate SNV data. The demographic characteristics of these 358 SKCM-TCGA patients were largely comparable to our cohort, whereby (n=220, 62%) were male and the median age was 57 years (95% CI 47-71 years) (**Supplementary Table 2**). The majority of these samples (n=283, 79%) were classified as 'metastasis', as opposed to 'primary' (n=75, 21%). The tumour sites were mainly from skin, subcutaneous or nodal metastatic sites, including 144 (40%) classified as from 'extremities', 134 (37%) from 'truncal' locations, 26 (7%) from the 'head and neck', 23 (6%) were 'regional lymph nodes' and the remainder were from other (less frequent) metastatic sites. Only 5 tumour samples in SKCM-TCGA were classified as from the 'brain' and these were excluded when we subsetted to the 'extracranial' metastatic melanoma comparator (n=274).

The MSK-IMPACT dataset was extracted from the publication by Zehir *et al*[11]. Samples were also filtered to a single sample per patient giving a total of 186 samples from 186 patients, all of which had appropriate SNV data. These samples were all labelled as 'cutaneous melanomas', and the majority (n=161, 87%) were classified as 'metastasis' as opposed to 'primary' (n=25, 13%). All primary tumours in this dataset (n=25) were classified as from 'skin' whereas the majority of metastatic samples were from 'regional lymph node' (n=25, 16%), 'lung' (n=24, 15%) and 'in-transit' (n=17, 11%) and the remainder were from other (less frequent) metastatic sites.

Both SKCM-TCGA and SKCM-MSK-IMPACT datasets included only cutaneous melanomas, in particular any melanomas within these datasets from acral, mucosal and other rarer sites were excluded. All SNVs reported from these datasets were non-synonymous mutations. We utilized the mutation calls provided by these resources and did not perform any additional filtering. The somatic mutational rate was calculated as the number of non-synonymous mutations divided by 30Mb, assuming that an average exome has 30Mb in protein-coding genes with sufficient coverage.

Copy number profiling of the SKCM-TCGA cohort

Copy number calls were generated from single nucleotide polymorphism (SNP data) within the SKCM-TCGA dataset using allele-specific copy number analysis (ASCAT version), as previously described.[12]

Statistical methods

All statistical analysis and graphics were generated using R version 3.5.3 (R Foundation for Statistical Computing, Vienna, Austria. URL http://www.R-project.org/).

Tests of equality of proportions: Logistic regression Wald t-tests (function glm) were used to test the equality of mutational frequencies in our cohort and in the reference datasets. Similar conclusions were obtained when considering Chi-square tests (function chisq.test in R) and Fisher's exact tests (function fisher.test in R). Furthermore, Monte Carlo simulations and considering scenarios corresponding to the observed numbers of successes and failures and based on 25,000 samples showed that the type I error of the chosen statistical methods lies between 0.02 to 0.03 instead of 0.05, suggesting that the conclusions drawn in the results are conservative.

Survival analyses: Overall survival from resection of brain metastasis was defined as the time from the date of resection of the brain metastasis to last follow-up (right-censored) or death from any cause. Overall survival from primary tumour was defined as the time from the date of resection of the primary tumour to last follow-up (right-censored) or death from any cause. Kaplan-Meier survival curves were estimated using the R function surv.fit ('Survival' package version 3.1-11). Univariate and multivariate hazard ratios (HR), 95% confidence intervals (95% CI) and corresponding p-values were obtained by fitting Cox proportional hazards regression models by means of the function coxph ('Survival' package version 2.4.2.). The multivariate analyses for overall survival from resection of brain metastases was controlled for sex, age at resection of brain metastasis, *BRAF* and *NRAS* mutation status as well as centre. Multivariate analyses for overall survival from primary tumour controlled for all the above, as well as primary tumour depth and ulceration. Multivariate analyses in TCGA dataset controlled for sex, age, *BRAF* and *NRAS* mutation status, Neoplasm disease stage as well as the number of non-synonymous mutations. Model check consist of proportional hazard chi-square tests by means of the function

cox.zph ('Survival' package), assessment of the c-statistics defined by means of the function concordance ('Survival' package), and deviance and Schoenfeld residual analyses obtained by means function ggcoxdiagnostics ('survminer' package, version 0.4.6).

Supplementary data

All the whole exome and targeted sequencing data (including raw sequencing files, variant calls and copy number calls) have been deposited at the European Genome-Phenome Archive (https://www.ebi.ac.uk/ega/ at the EBI) under study accession ID EGAS00001002107. Each individual dataset is available under the following dataset accession ID's:

Discovery cohort raw whole-exome sequencing files: EGAD00001005981

Discovery cohort variant calls: EGAD00001005982

Discovery cohort copy number calls: EGAD00001005983

Orthogonal validation for discovery cohort, raw sequencing files: EGAD00001005984

External validation cohort, raw sequencing files: EGAD00001005985

Supplemental Table Legends

Supplementary Table 1. Demographic and clinical characteristics of all samples in the discovery cohort. The table also includes the matched germline samples, as well as the details from three extracranial samples relating to this cohort (the latter with concordant *KRAS* mutations). These are indicated in the column "Tissue type". Cambridge: Cambridge University Hospitals, MIA: Melanoma Institute of Australia, Queensland: The University of Queensland Australia. SSM: Superficial spreading melanoma, NM: Nodular melanoma, LMM: Lentigo-maligna melanoma.

Supplementary Table 2. Demographic and clinical characteristics of all samples in the SKCM-TCGA cohort.

Supplementary Table 3. List of cancer driver genes (n=549) included in the custom capture bait used on the (n=18) external validation samples.

Supplementary Table 4. List of cancer driver genes (n=278) included in the custom capture bait used on the orthogonal validation of driver SNVs (n=60) from the discovery cohort.

Supplementary Table 5. Clinical and mutational characteristics of the patients with hotspot *KRAS* mutations (including both patients from the discovery and validation cohorts). Of note all hotspot *KRAS* mutations were identified in patients with either thin (T1/T2) or no prior history of primary melanoma, and from primary tumours in chronically sun-exposed locations. Mutations in *KRAS* also had a high variant allele frequency, indicating that these likely represent clonal driver mutations, which is further supported by their concordance in extracranial metastases.

Supplementary Table 6. Clinical and mutational characteristics of the patients with hotspot *KRAS* mutations in SKCM-TCGA (n=6) and SKCM-MSK-IMPACT (n=2). *KRAS* mutations were mutually exclusive to *BRAF/NRAS/HRAS* hotspot mutations in both these datasets.

Supplementary Table 7. Clinical characteristics of hotspot *KRAS*-mutant (n=4) vs *KRAS*-wild type (n=46) patient within the discovery cohort.

References

1. Li H. Aligning sequence reads, clone sequences and assembly contigs with BWA-MEM. *ArXiv* **1303** (2013).
2. Cibulskis K, Lawrence MS, Carter SL, Sivachenko A, Jaffe D, Sougnez C *et al.* Sensitive detection of somatic point mutations in impure and heterogeneous cancer samples. *Nat Biotechnol* **31**, 213-219 (2013).
3. Saunders CT, Wong WS, Swamy S, Becq J, Murray LJ, Cheetham RK. Strelka: accurate somatic small-variant calling from sequenced tumor-normal sample pairs. *Bioinformatics* **28**, 1811-1817 (2012).
4. Chen X, Schulz-Trieglaff O, Shaw R, Barnes B, Schlesinger F, Kallberg M *et al.* Manta: rapid detection of structural variants and indels for germline and cancer sequencing applications. *Bioinformatics* **32**, 1220-1222 (2016).
5. McLaren W, Gil L, Hunt SE, Riat HS, Ritchie GR, Thormann A *et al.* The Ensembl Variant Effect Predictor. *Genome Biol* **17**, 122 (2016).
6. Lek M, Karczewski KJ, Minikel EV, Samocha KE, Banks E, Fennell T *et al.* Analysis of protein-coding genetic variation in 60,706 humans. *Nature* **536**, 285-291 (2016).
7. Favero F, Joshi T, Marquard AM, Birkbak NJ, Krzystanek M, Li Q *et al.* Sequenza: allele-specific copy number and mutation profiles from tumor sequencing data. *Ann Oncol* **26**, 64-70 (2015).
8. Genomic Classification of Cutaneous Melanoma. *Cell* **161**, 1681-1696 (2015).
9. Cerami E, Gao J, Dogrusoz U, Gross BE, Sumer SO, Aksoy BA *et al.* The cBio cancer genomics portal: an open platform for exploring multidimensional cancer genomics data. *Cancer discovery* **2**, 401-404 (2012).
10. Gao J, Aksoy BA, Dogrusoz U, Dresdner G, Gross B, Sumer SO *et al.* Integrative analysis of complex cancer genomics and clinical profiles using the cBioPortal. *Science signaling* **6**, pl1 (2013).
11. Zehir A, Benayed R, Shah RH, Syed A, Middha S, Kim HR *et al.* Mutational landscape of metastatic cancer revealed from prospective clinical sequencing of 10,000 patients. *Nat Med* **23**, 703-713 (2017).
12. Van Loo P, Nordgard SH, Lingjærde OC, Russnes HG, Rye IH, Sun W *et al.* Allele-specific copy number analysis of tumors. *Proc Natl Acad Sci U S A* **107**, 16910-16915 (2010).

Supplementary Figure 1

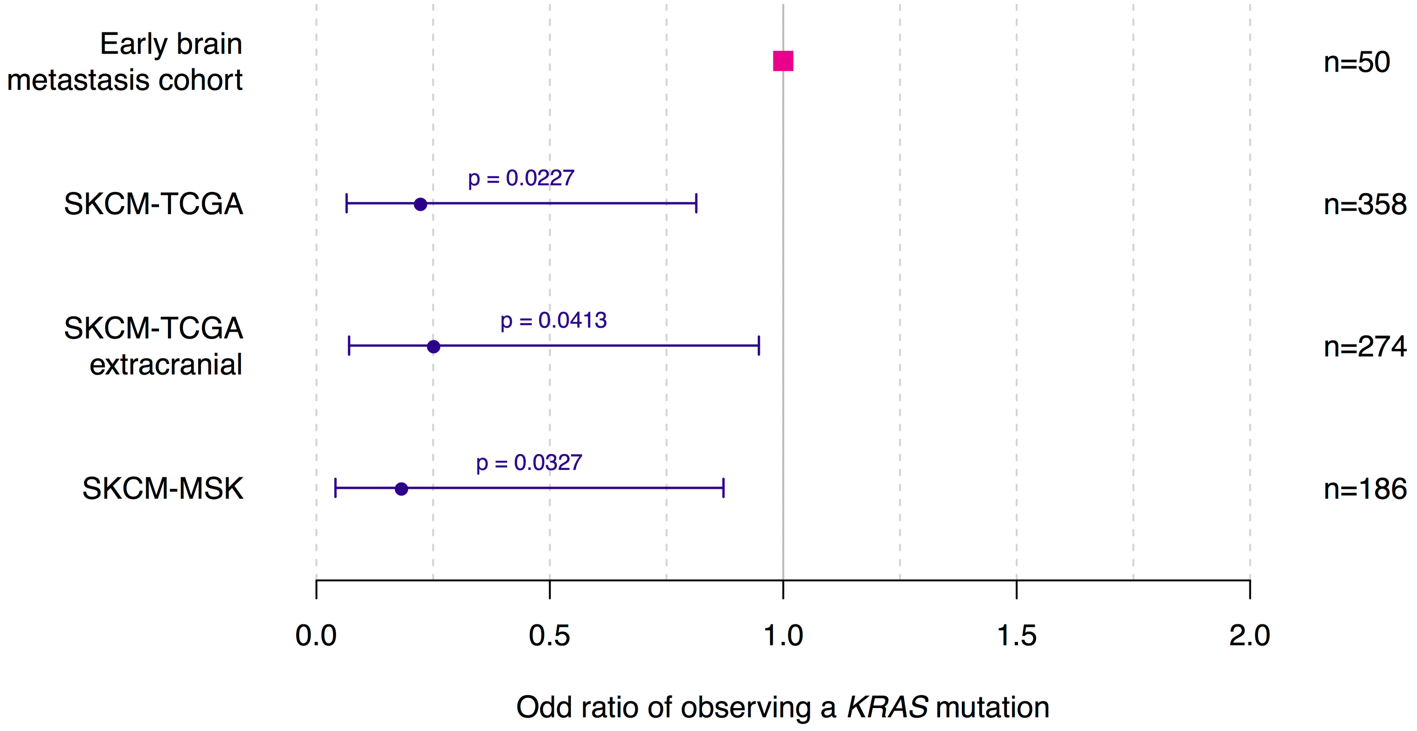

Supplementary Fig. 1. Odds ratio plot showing the odds of observing a *KRAS* mutation in the three reference datasets relative to the early brain metastases discovery cohort (baseline). Dots correspond to logistic regression odd ratio estimates and range to the corresponding 95% confidence interval. The odds of observing a *KRAS* mutation in a given sample within the discovery cohort was ~4-fold higher than in these three reference datasets, this difference is significant.

Supplementary Figure 2

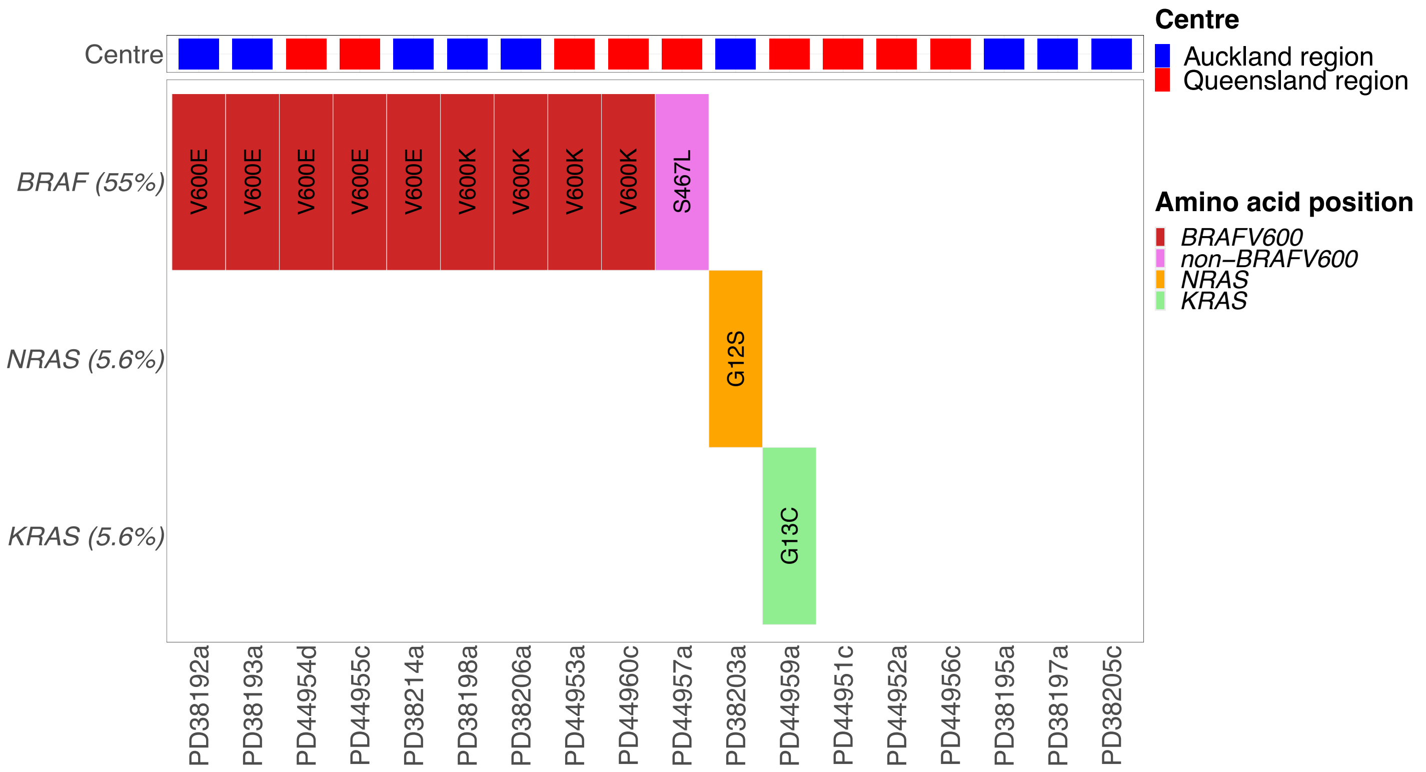

Supplementary Fig. 2. Tile plot of mutated positions within the RAS signalling genes in the external validation cohort (n=18). The *KRAS*^{G13C} mutation was mutually exclusive from both *BRAF* and *NRAS* hotspot mutations, in keeping with the findings from the discovery cohort (**Fig. 1B**).

Supplementary Figure 3

A

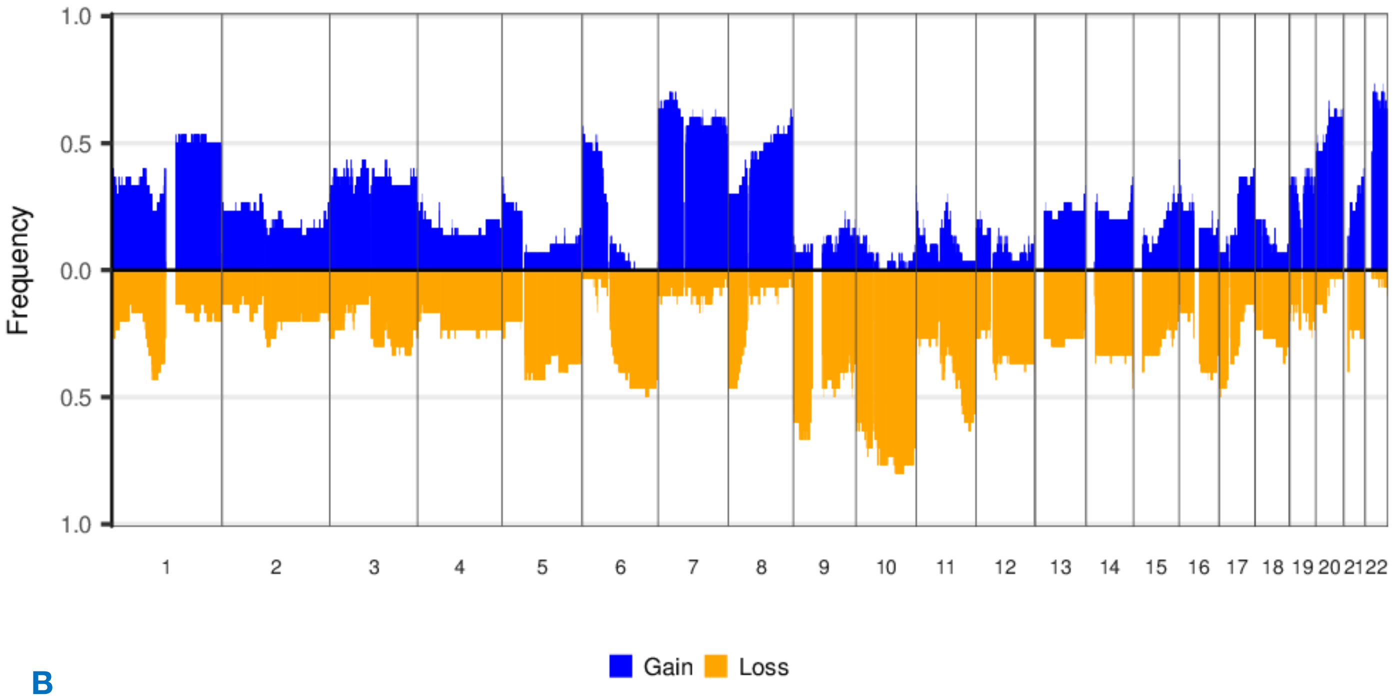

B

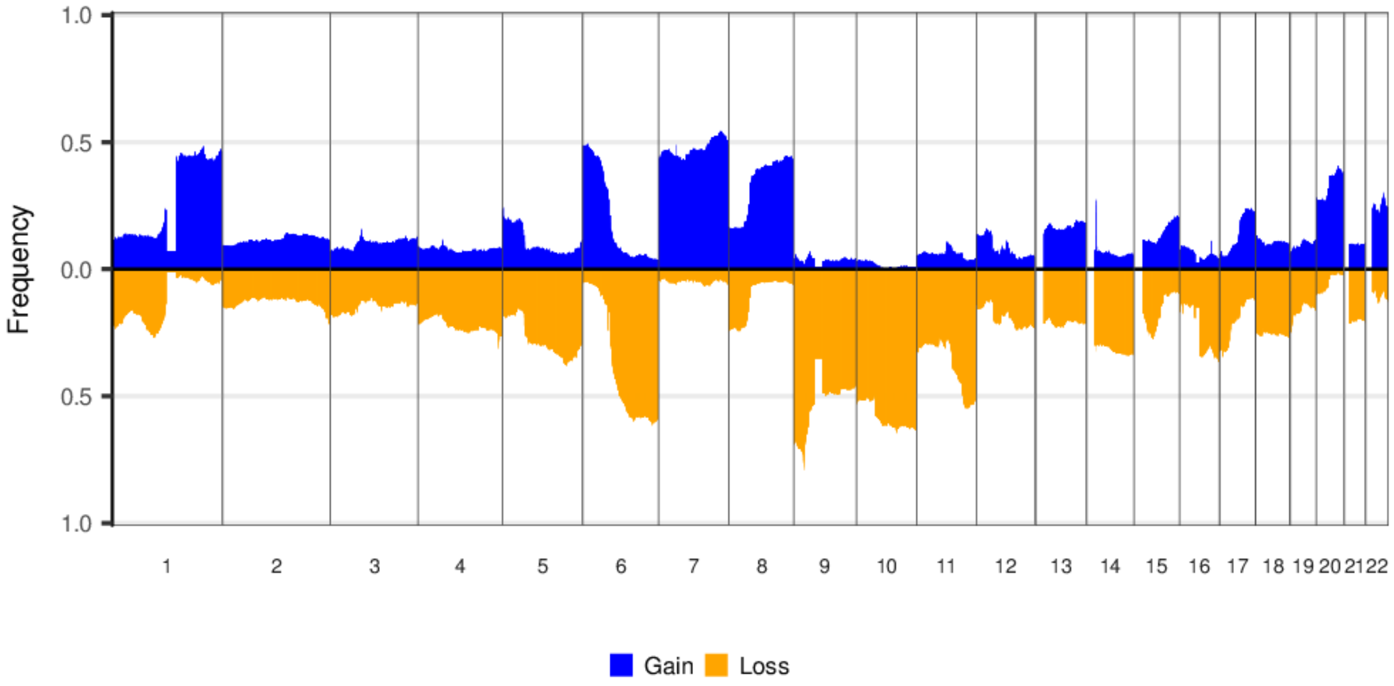

Supplementary Fig. 3. Copy number profile of (A) Early melanoma brain metastases from the discovery cohort (n=30) and (B) Cutaneous melanomas from The Cancer Genome Atlas (SKCM-TCGA, n=337).

Supplementary Figure 4

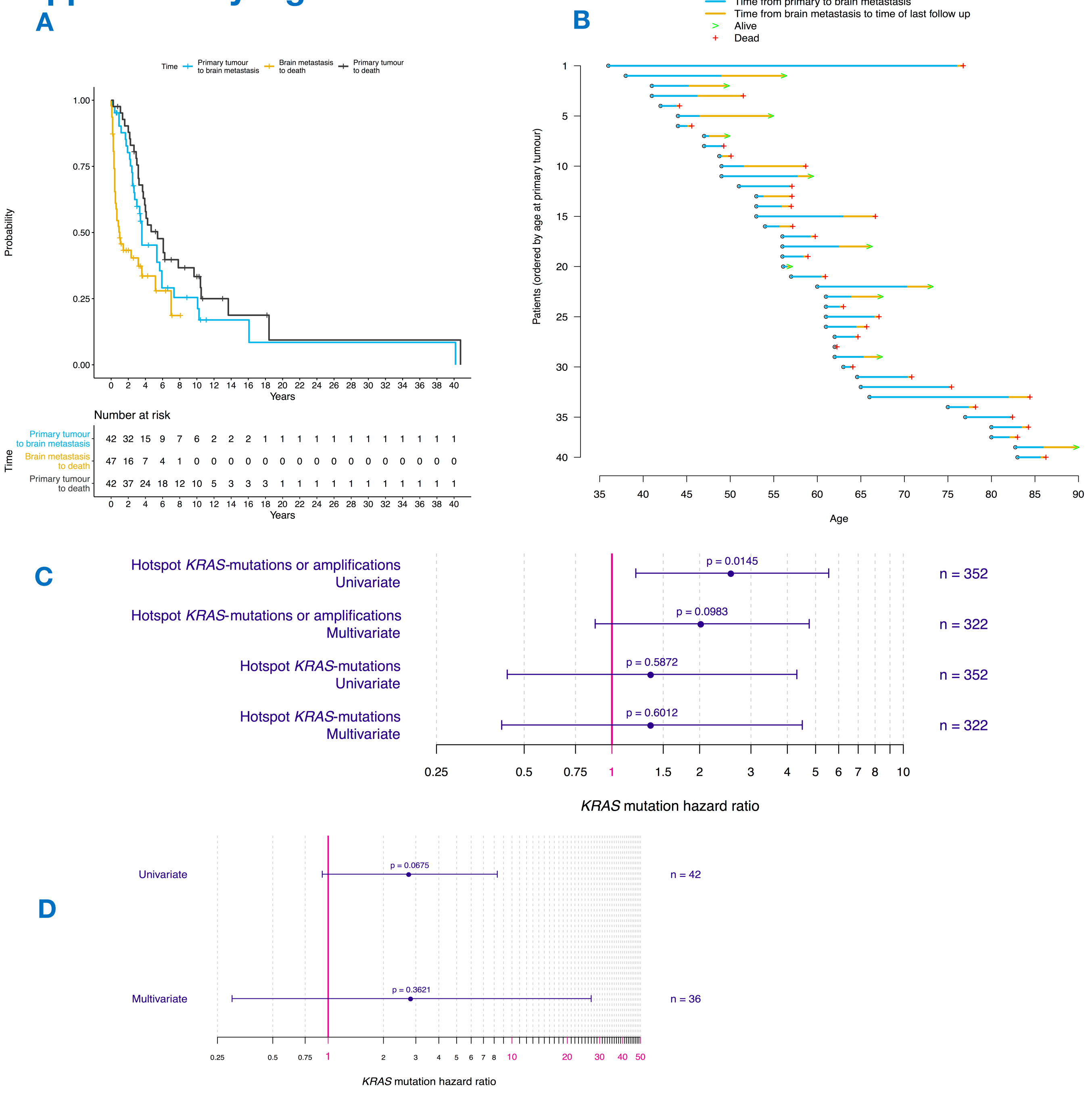

Supplementary Fig. 4. Survival plots of the early brain metastasis discovery cohort. A) Kaplan-Meier survival plots showing, Black line: survival from primary disease (defined as the time from the resection of the primary tumour to last follow-up (censored) or death from any cause, indicated in black) as a function of time (median survival from primary disease 65.0 months, 95% CI 45.0-125.0 months). Survival data from primary disease was only available on 42 patients (3 patients had no history of primary melanoma, 5 patients had no survival information). Orange line: survival from resection of brain metastasis, defined as the time from the resection of brain metastasis to last follow-up (censored) or death from any cause as a function of time. Median 12.0 months, 95% CI 6.0-62.0 months. Data from resection of brain metastasis was available on 47 patients. Blue line: Time to progression from primary tumour to brain metastases. **B)** Timeline summary for (n=40) patients. Patients are ordered according to the age at primary tumour resection (y-axis). **C)** Impact of *KRAS* mutational status on overall survival from primary tumour as a function of time. **D)** Forest plot comparing *KRAS*-mutant versus *KRAS*-wild-type overall survival from primary tumour in univariate (HR 2.78, 95% CI 0.93-8.32, p=0.0675, n=42) and multivariate (HR 2.85, 95% CI 0.30-26.97, p=0.3621, n=36) Cox proportional hazards regression models. Multivariate correction was undertaken for gender, centre, age at primary tumour resection, T-stage and ulceration of primary tumour, as well as *BRAF* and *NRAS* mutation status.

Supplementary Figure 5

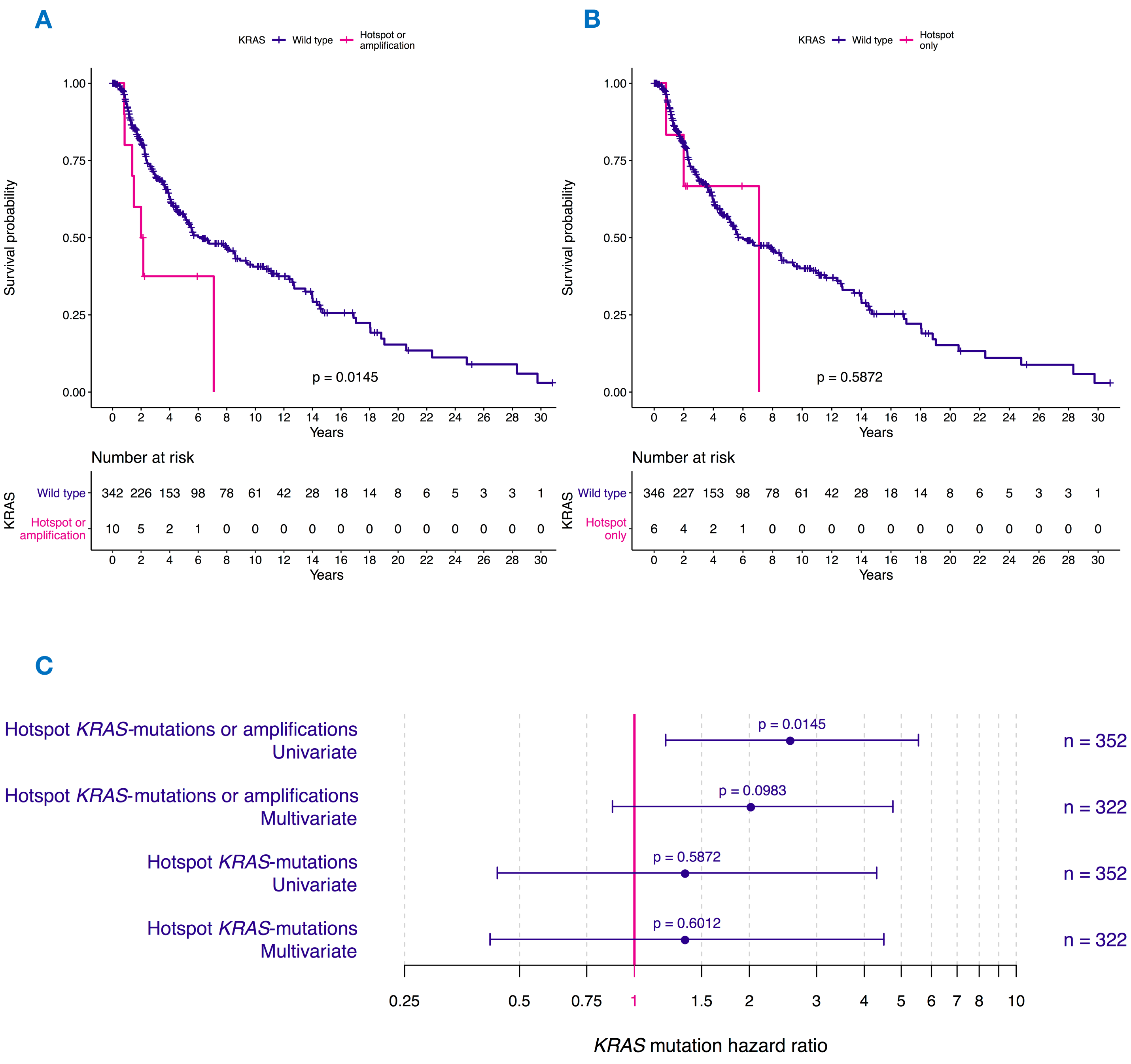

Supplementary Fig. 5. Impact of *KRAS* mutational status on overall survival in SKCM-TCGA. A) Kaplan-Meier curves for overall survival in *KRAS* hotspot mutations or amplifications (n=10) versus *KRAS*-WT (n=342) melanoma in SKCM-TCGA. **B)** Overall survival in *KRAS* hotspot mutation only (n=6) versus *KRAS*-WT (n=346) melanoma in SKCM-TCGA. **C)** Forest plot showing *KRAS*-mutant survival in SKCM-TCGA (hotspot mutations or amplifications) in univariate (HR 2.59, 95% CI 1.21-5.55, p=0.015, n=352) and multivariate analyses (HR 2.04, 95% CI 0.88-4.75, p=0.098, n=322). As well *KRAS*-mutant survival in SKCM-TCGA (hotspot mutations alone) in univariate (HR 1.37, 95% CI 0.44-4.31, p=0.587, n=352) and multivariate analyses (HR 1.37, 95% CI 0.41-4.50, p=0.601, n=322). Generated using Cox proportional hazards regression models. Multivariate correction was undertaken for stage, sex, age at diagnosis of primary, non-synonymous mutation count as well for *BRAF* and *NRAS* mutation status.
